# Exertional Rhabdomyolysis Following Concurrent Exercise Including Pelvic Floor (Kegel) Training in a Previously Healthy 36-Year-Old Male Patient: A Case Report

**DOI:** 10.7759/cureus.111914

**Published:** 2026-07-01

**Authors:** Brooke Kelly, Karen Lazarus, Jordan Knox

**Affiliations:** 1 Internal Medicine, Sidney Kimmel Medical College, Philadelphia, USA; 2 Family Medicine, Thomas Jefferson University, Philadelphia, USA

**Keywords:** case report, creatine kinase, kegels, myoglobinuria, pelvic floor, rhabdomyolysis

## Abstract

Rhabdomyolysis is a potentially life-threatening condition characterized by skeletal muscle breakdown and release of intracellular contents into the systemic circulation. Exertional rhabdomyolysis (ER) most commonly occurs after high-intensity exercise involving large muscle groups, but cases related to lower intensity or repetitive activity have also been reported. We describe a case of acute ER in a previously healthy 36-year-old man who developed the condition after initiating a workout regimen for erectile dysfunction that included gluteal exercises and pelvic floor muscle training (PFMT), including Kegel exercises, demonstrated in YouTube (Google, San Bruno, CA) videos. The patient presented with dark, reddish-brown urine and mild soreness in the hips and lower back several days after a workout consisting of repetitive pelvic floor contractions, glute bridges, and pelvic tilts. He noted that he had difficulty walking two days after completing the exercises, which had resolved by the time of his visit. Physical examination was unremarkable; however, laboratory testing revealed markedly elevated creatine kinase levels (>22,000 U/L) with associated transaminase elevation. Urinalysis demonstrated positive blood on dipstick without microscopic hematuria, consistent with myoglobinuria. The patient was admitted for aggressive intravenous hydration and monitored closely, with gradual improvement in laboratory values during a four-day hospital course and full recovery without renal complications. This case highlights an uncommon presentation of ER following initiation of an unsupervised exercise regimen that included PFMT, along with gluteal and core exercises. It illustrates that even exercises generally considered lower impact may contribute to significant muscle injury when performed without appropriate instruction and gradual conditioning or in previously unaccustomed individuals. Awareness of atypical triggers and subtle clinical presentations may help clinicians recognize rhabdomyolysis earlier and initiate prompt treatment to prevent serious complications.

## Introduction

Rhabdomyolysis is a pathophysiologic condition characterized by severe injury to muscle tissue, leading to the subsequent release of intracellular contents into the circulation, including electrolytes, myoglobin, and sarcoplasmic proteins. Clinical definitions of rhabdomyolysis vary, but typically include a syndrome of acute muscle weakness, myalgia, and muscle swelling combined with a creatine kinase (CK) cutoff value of >1,000 U/L or CK greater than five times the upper limit of normal [1,2]. While there is no consensus regarding the most accurate way to classify rhabdomyolysis, there have been eight major etiologies identified, including trauma, muscle hypoxia, genetic defects, infections, body-temperature changes, metabolic and electrolyte disorders, drugs and toxins, and exertion [3].

Exertional rhabdomyolysis (ER) is often caused by high-intensity exercise, eccentric exercises, or sudden modifications to training. Other factors that amplify risk for ER include dehydration, heat illness, and poor conditioning [4]. Certain individual characteristics and exposures have also been associated with increased risk, including sickle cell trait, certain single nucleotide polymorphisms, use of medications such as statins or anticholinergics, and dietary supplements such as caffeine [5-7].

In ER, stretching or intense muscle activity triggers the influx of sodium, chloride, and water into muscle cells, resulting in swelling and breakdown. Simultaneously, calcium influx triggers persistent contraction, energy depletion, protease activation, and free-radical production. Neutrophil-mediated inflammation ensues, further perpetuating muscle tissue disintegration and release of myocyte contents [3,8]. While many cases of rhabdomyolysis are mild, accumulation of these cellular components within the systemic circulation can have serious consequences, including electrolyte abnormalities, compartment syndrome, disseminated intravascular coagulation, cardiac arrhythmias, acute kidney injury, and renal failure [3,5,8].

Typical presentations of ER involve severe muscle pain, swelling, and weakness on both active and passive motion. Patients might experience nausea or fever and may describe dark urine color [5,9]. Muscle soreness, weakness, and swelling will typically arise within the first 72 hours of performing activity that is extreme, prolonged, or unfamiliar [9].

While this phenomenon is well described in athletes and individuals performing high-intensity exercise, ER resulting from lower intensity or isolated muscle training is rarely reported. According to the American College of Sports Medicine exercise prescription guidelines, vigorous-intensity activity corresponds to 60%-89% of heart rate reserve (HRR) or VO₂ reserve (VO₂R), whereas moderate-intensity activity corresponds to 40%-59% of HRR or VO₂R. Low-intensity activity is generally defined as occurring below these thresholds [10].

Pelvic floor muscle training (PFMT) is a conservative, noninvasive therapy commonly used for male erectile and urinary dysfunction and is typically considered a low-load form of exercise. These exercises may improve erectile function by strengthening the bulbospongiosus and ischiocavernosus muscles, which contribute to penile rigidity and venous occlusion [11]. Pelvic floor exercises consist of repetitive, isolated contractions that may be performed with verbal instruction, biofeedback, or supervised physical therapy [11-13]. Although these exercises involve relatively small muscle groups, repetitive unaccustomed contractions may still result in localized muscle injury and myocyte breakdown through the same mechanisms implicated in ER of larger skeletal muscle groups [12]. Proper technique requires isolation of the pelvic floor musculature without recruitment of the abdominal, gluteal, or adductor muscles, which can make both instruction and execution challenging [11,13,14]. When performed correctly, pelvic floor training is generally considered safe and well tolerated. ER following isolated lower body and pelvic-floor exercises has rarely been reported [15-19].

Here, we present a clinical case of ER occurring after initiation of a workout regimen that included gluteal, core, and pelvic floor exercises in an otherwise healthy 36-year-old man.

## Case presentation

A 36-year-old African American man with a past medical history of attention-deficit/hyperactivity disorder (ADHD) and major depressive disorder (MDD) presented to his primary care provider for sexual dysfunction. He reported distressing symptoms of at least three months of difficulty maintaining erections, reduced firmness of erections, and premature ejaculation. He was prescribed tadalafil 5 mg daily with an additional 5 mg dose as needed for sexual activity. He did not receive guidance regarding specific exercises, proper technique, recommended volume, or training frequency, nor was he referred for formal physical therapy. At the time, his only regular physical activity consisted of the occupational demands of his work as a plumber.

Two weeks later, the patient returned to his primary care provider with concern for blood in his urine. For two days, he had noticed reddish-brown urine that lightened after hydration. He also reported soreness in his hips, legs, and lower back. He attributed these symptoms to a single workout session he performed earlier that week to alleviate symptoms of erectile dysfunction, as recommended during his previous visit. The patient followed two YouTube (Google, San Bruno, CA) workout videos involving bodyweight exercises targeting the pelvic floor and surrounding muscle groups. The first video instructed viewers to perform 100 repetitions each of butterfly beats, diamond leg raises, glute bridges, and kneeling hip thrusts, for a total of approximately 400 repetitions. The second video consisted of a seven-minute circuit that included 10 repetitions each of several exercises, including glute bridge variations, scissor kicks, planks, adductor extensions, squats, and single-leg lunges. Participants were encouraged to take only brief rest periods of 5-10 seconds between exercises, with a single 20-second break midway through the routine for stretching. The patient reported completing the first routine and repeating the second routine multiple times during a single exercise session lasting slightly more than one hour. He stated that he remained adequately hydrated throughout the workout and took occasional short breaks between sets. Neither video included a warm-up or cool-down period. He did not perform any additional exercise before presentation and reported that he had been largely inactive for several weeks prior to this session.

The patient denied symptoms other than muscle soreness. He denied any dysuria, burning, or frequency. He denied fever, chills, nausea, vomiting, or systemic symptoms. He had no history of kidney stones, urinary tract infection, or hematuria in the past. He had no changes in vision or hearing, no chest pain, shortness of breath, or chest pressure. He had no numbness, weakness, dizziness or syncope, or neurologic symptoms.

His only past medical history included MDD and ADHD. At the time, he was taking no medications other than prescribed tadalafil. He was also taking supplements including fish oil and vitamins C, D, E, and K. He drank two alcoholic beverages a week. He reported daily cannabis use, smoking approximately three joints per day. He has never used tobacco products or other substances.

No abnormalities were noted upon physical examination. The patient appeared comfortable and had no muscle swelling or tenderness to palpation in his hips or upper thighs. His strength was intact in upper and lower extremities bilaterally.

Acute cystitis and nephrolithiasis were initially considered given the patient’s discolored urine. However, the absence of dysuria, urinary frequency, flank pain, fever, or significant hematuria on microscopy made these diagnoses less likely. The combination of recent exertional activity, urine dipstick positivity for blood without red blood cells on microscopy, and markedly elevated CK levels supported rhabdomyolysis as the primary diagnosis. Due to concern for rhabdomyolysis, the patient was sent directly to the emergency department for further evaluation. Laboratory testing in the emergency department revealed a CK level greater than 22,000 U/L and elevated liver enzymes (aspartate aminotransferase (AST) = 1,251 U/L, alanine aminotransferase (ALT) = 306 U/L). Additional laboratory findings obtained on admission are summarized in Table 1. Notably, renal function remained preserved, with a creatinine of 1.07 mg/dL and normal electrolyte levels, while urinalysis demonstrated positive blood on dipstick with fewer than one red blood cell per high-power field, consistent with myoglobinuria (Table 1).

**Table 1:** Admission laboratory findings in a patient with suspected exertional rhabdomyolysis Results are notable for markedly elevated creatine kinase and transaminases, with urinalysis demonstrating heme positivity in the absence of significant red blood cells, consistent with myoglobinuria CBC: complete blood count; eGFR: estimated glomerular filtration rate; HPF: high-power field; MCH: mean corpuscular hemoglobin; MCHC: mean corpuscular hemoglobin concentration; MCV: mean corpuscular volume; MDMA: methylenedioxymethamphetamine; MPV: mean platelet volume; pCO₂: partial pressure of carbon dioxide; PCP: primary care provider; RBC: red blood cell; RDW: red cell distribution width; WBC: white blood cell

Test	Normal range	Value on admission day
Routine chemistry		
Sodium (mmol/L)	135-145	139
Potassium (mmol/L)	3.5-5.0	4
Chloride (mmol/L)	98-106	102
pCO₂ (mmHg)	35-45	27
Anion gap (mmol/L)	8-12	10
Urea nitrogen (mg/dL)	7-20	14
Creatinine (mg/dL)	0.6-1.3	1.07
eGFR (mL/min/1.73 m²)	>60	93
Calcium (mg/dL)	8.6-10.2	9.1
Magnesium (mg/dL)	1.7-2.2	1.7
Total protein (g/dL)	6.0-8.3	7.1
Albumin (g/dL)	3.5-5.0	4.4
Bilirubin, total (mg/dL)	0.1-1.2	1.4
Bilirubin, direct (mg/dL)	0-0.3	0.3
Alkaline phosphatase (U/L)	44-147	68
Alanine transaminase (U/L)	7-56	306
Aspartate aminotransferase (U/L)	10-40	1,251
Creatine kinase (U/L)	30-200	>22,000
CBC		
Hemoglobin (g/dL)	12-16	14
Hematocrit (%)	36-46	41.2
WBC (×10⁹/L)	4.0-11.0	4.6
RBC (×10¹²/L)	4.0-5.5	4.42
MCV (fL)	80-100	93
MCH (pg)	27-33	31.7
MCHC (g/dL)	32-36	34
RDW (%)	11.5-14.5	13.7
MPV (fL)	7.5-11.5	11.3
Urinalysis		
Color	Yellow	Yellow
Appearance	Clear	Clear
Specific gravity, urine	1.005-1.030	1.027
pH, urine	4.5-8.0	6.5
Protein, urine	Negative	1+
Glucose, urine	Negative	Negative
Bilirubin, urine	Negative	Negative
Ketones, urine	Negative	1+
Blood, urine	Negative	2+
Nitrite, urine	Negative	Negative
Urobilinogen, urine (EU/dL)	0.2-1.0	1+
Leukocyte esterase	Negative	Negative
RBC, urine (per HPF)	0-2	<1
WBC, urine (per HPF)	0-5	1
Mucous threads	None-rare	Rare
Toxicology, urine		
Amphetamine	Negative	Negative
Barbiturate	Negative	Negative
Benzodiazepine	Negative	Negative
Cannabinoid	Negative	Positive
Cocaine	Negative	Negative
Fentanyl	Negative	Negative
Methadone	Negative	Negative
Opiate	Negative	Negative
Oxycodone	Negative	Negative
Phencyclidine	Negative	Negative
Propoxyphene/MDMA	Negative	Negative

**Figure 1:**
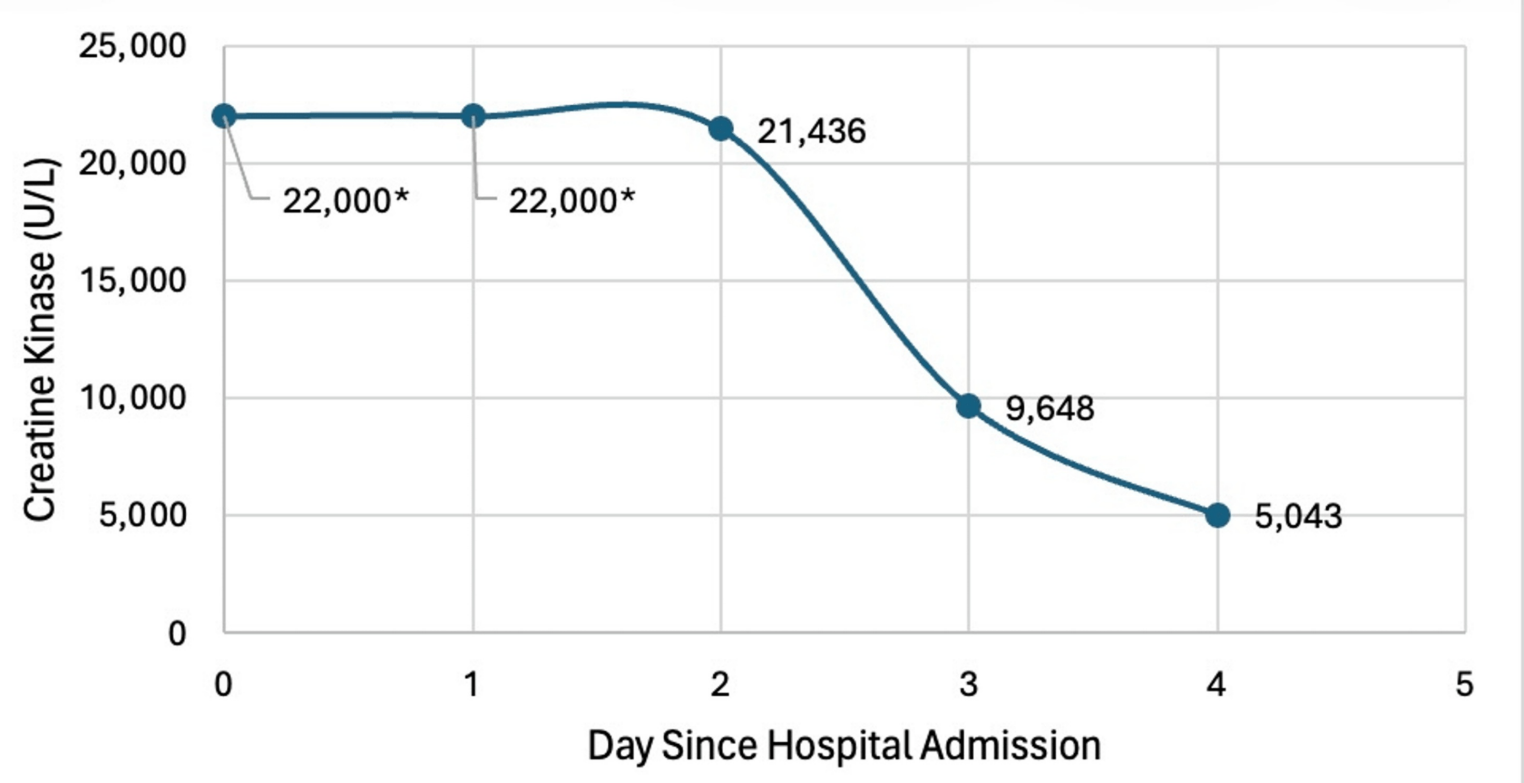
Trend in CK levels during hospitalization for exertional rhabdomyolysis Serial CK measurements demonstrating the trajectory of muscle injury and recovery following admission. CK levels peaked at >22,000 U/L and declined steadily with intravenous fluid resuscitation ^*^Day 0 CK value reported as >22,000 U/L CK: creatine kinase

**Figure 2:**
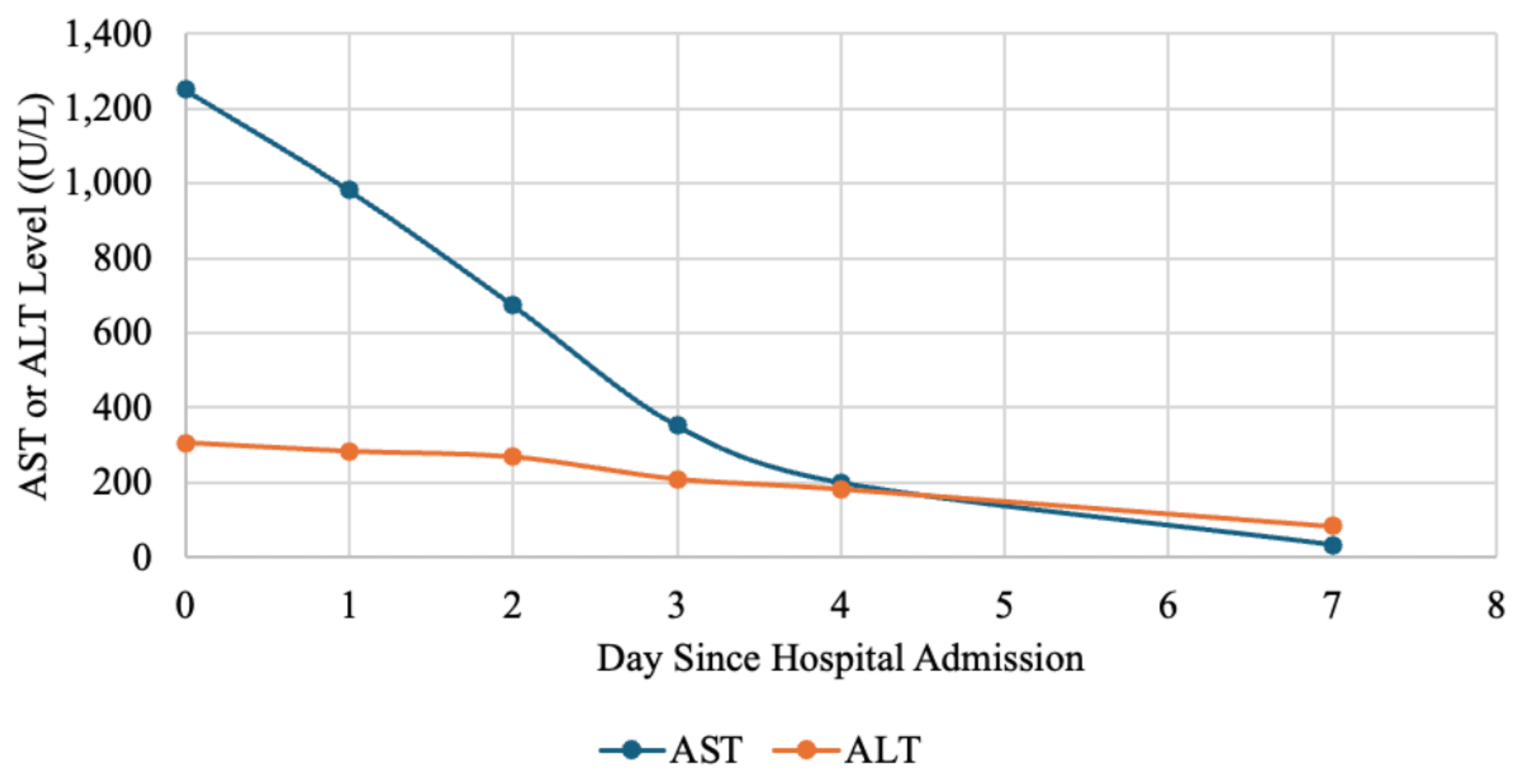
Trends in AST and ALT levels during hospitalization Serial measurements of AST and ALT demonstrating improvement in transaminase elevations associated with rhabdomyolysis over the course of hospitalization and treatment AST: aspartate aminotransferase; ALT: alanine aminotransferase

Given the abnormalities in CK and liver enzymes, the patient was subsequently admitted to the hospital for treatment in line with general consensus treatment for rhabdomyolysis [3,20]. He was given normal saline at 250 mL/hour with a goal urine output of 200-300 mL/hour for three days. The laboratory trends demonstrated in Figures 1, 2 illustrate the patient’s response to treatment throughout his hospital course. As shown in Figure 1, CK levels declined steadily following initiation of intravenous fluid resuscitation, consistent with resolution of skeletal muscle injury. Similarly, Figure 2 demonstrates progressive improvement in AST and ALT levels over the course of hospitalization.

The patient was discharged on hospital day 4 following gradual correction of his laboratory abnormalities and improvement in clinical symptoms. The downward trends in CK, AST, and ALT observed during hospitalization (Figures 1, 2) continued after discharge, with CK normalizing and AST and ALT improving to 34 and 83 U/L, respectively, at outpatient follow-up. The patient did not receive formal return-to-activity guidance at discharge and was advised only to resume activity as tolerated while monitoring for recurrent symptoms, including muscle pain, weakness, or dark urine. He refrained from structured exercise for approximately two weeks following discharge. At that time, after complete resolution of muscle soreness and normalization of urine color, he resumed exercise three times weekly. Rather than following a graded rehabilitation program, he returned directly to a full-body resistance-training regimen consisting of exercises he had previously learned from personal trainers and online workout videos. At follow-up with his primary care provider, he reported complete resolution of symptoms and no recurrence of muscle soreness, pigmenturia, or other complications after resuming exercise.

## Discussion

ER most commonly occurs following high-intensity or unfamiliar physical activity involving large muscle groups. Common precipitating activities include marathon running, soccer, body-building, military training, and CrossFit [5,16,20]. During vigorous or repetitive muscle contraction, disruption of myocyte membranes releases intracellular contents, including CK, myoglobin, and electrolytes, into the circulation [3,8,16]. Marked elevation of AST and ALT may also occur in rhabdomyolysis due to release of transaminases from injured skeletal muscle, which can initially raise concern for a primary hepatic process [21-23]. In the case above, repetitive contraction of previously unconditioned pelvic floor, gluteal, and core musculature during an unfamiliar exercise regimen likely contributed to metabolic stress, resulting in muscle fiber injury and rhabdomyolysis.

Although ER is typically associated with large muscle groups, smaller and less commonly exercised muscle groups may also be affected under certain conditions. PFMT, including Kegel exercises, involves the repeated voluntary contraction of muscles such as the striated urethral sphincter, levator ani, bulbocavernosus, and ischiocavernosus [11,13-15]. PFMT is commonly recommended as a nonpharmacologic intervention for urinary incontinence and sexual dysfunction in men [11,13-15]. These exercises are generally considered safe and low-impact when performed with appropriate conditioning and gradual progression. They also primarily involve concentric muscle contractions, which are less frequently associated with rhabdomyolysis compared with eccentric exercises [4].

In the present case, the patient performed a high volume of repetitive pelvic floor contractions, pelvic tilts, glute bridges, kneeling hip thrusts, squats, and lunges despite limited prior structured exercise activity outside of occupational physical labor. While PFMT was the original motivation for the workout, several of the exercises performed also recruited larger muscle groups, including the gluteal, hamstring, quadriceps, hip adductor, and core musculature. Glute bridges and hip thrusts, in particular, involve repeated activation of the gluteal and posterior chain muscles and therefore engage substantially greater muscle mass than isolated pelvic floor contractions [12]. Accordingly, the marked CK elevation observed in this patient likely reflects the cumulative effect of repetitive contractions across multiple previously unconditioned muscle groups rather than isolated pelvic floor contractions alone.

These findings are consistent with prior reports of ER following other repetitive or unfamiliar exercise regimens, including yoga, spin classes, push-up challenges, sit-ups, and other high-repetition activities [16-20]. Collectively, these cases suggest that ER is not solely determined by absolute exercise intensity but may also occur when repetitive contractions overwhelm the metabolic capacity of previously unconditioned muscle groups [5,24,25]. Although the specific muscle groups contributing most to the injury cannot be determined, repetitive contractions involving both pelvic floor and larger lower extremity muscle groups may have produced sustained calcium influx, adenosine triphosphate depletion, and oxidative injury, ultimately resulting in myocyte breakdown and rhabdomyolysis [3,12].

Beyond the unusual mechanism of injury, another notable aspect of this case was the patient's relatively mild clinical presentation despite severe biochemical evidence of muscle injury. As shown in Table 1, he presented with a CK level >22,000 U/L and marked aminotransferase elevations despite preserved renal function and minimal symptoms, highlighting the potential disconnect between clinical presentation and disease severity in ER. Elevated aminotransferases in rhabdomyolysis may arise from injured skeletal muscle or reflect concurrent hepatic injury secondary to protease release and oxidative stress. Prior studies have suggested that CK/ALT and CK/AST ratios may help distinguish muscle-derived enzyme elevations from primary liver injury, although definitive cutoff values have not been established [21,22]. In this patient, the CK/ALT and CK/AST ratios were 17.6 and 71.9, respectively, supporting skeletal muscle injury as the predominant source of aminotransferase elevation, though a mixed contribution from both muscle and hepatic injury cannot be excluded.

It is important to note that the classic triad of rhabdomyolysis, including myalgia, weakness, and dark urine, is present in less than 10% of patients [3]. In this case, the patient reported mild soreness and darkened urine but lacked significant muscle tenderness, swelling, or systemic symptoms. The absence of severe pain or weakness could potentially delay recognition of rhabdomyolysis, particularly when the precipitating activity is perceived as low risk. The patient’s urinalysis showing positive blood on dipstick without microscopic hematuria was consistent with myoglobinuria and helped prompt further evaluation.

In addition to demographic risk factors described in the literature, including male sex, African American race, and younger age, the present patient had several modifiable risk factors for ER, including recent inactivity, low baseline conditioning, and sudden participation in a prolonged unfamiliar exercise routine [5,9,23-25]. Notably, although the patient worked as a plumber and routinely performed physically demanding occupational tasks, his presentation highlights that occupational physical activity does not necessarily confer conditioning for high-repetition, targeted exercise involving specific muscle groups. Even individuals who are physically active through work may remain susceptible to ER when exposed to unfamiliar movement patterns or repetitive contractions involving previously untrained musculature.

Furthermore, the patient reported modest alcohol and significant cannabis use, both of which have been associated with rhabdomyolysis. Alcohol may contribute to muscle injury through direct myotoxic effects and through activation of inflammatory pathways during muscle recovery [26]. In many cases, however, rhabdomyolysis related to alcohol use occurs secondary to prolonged immobilization during intoxication rather than from direct muscle toxicity alone [26-28]. Chronic alcohol use may also predispose individuals to rhabdomyolysis by promoting electrolyte abnormalities such as hypokalemia and hypophosphatemia [29]. The patient also reported regular cannabis use, which was confirmed on urine toxicology at admission (Table 1). Cannabis has been linked to cases of rhabdomyolysis, although the underlying mechanism remains poorly understood [30-33]. Finally, the patient was also taking tadalafil for erectile dysfunction. Although phosphodiesterase-5 inhibitors possess vasodilatory properties, there is insufficient evidence to suggest that therapeutic tadalafil use meaningfully increases the risk of ER, and its contribution to the present case remains unclear.

An additional consideration in this case is the absence of a structured exercise prescription. Although the patient was encouraged to perform pelvic floor exercises for erectile dysfunction, he was not provided specific guidance regarding exercise frequency, intensity, duration, or progression. Exercise prescription frameworks, such as the Frequency, Intensity, Time, and Type principle, emphasize tailoring exercise recommendations to an individual's baseline conditioning and progressively increasing the workload over time [10]. In this case, the lack of specific exercise parameters may have contributed to the patient's decision to perform a prolonged, high-volume workout consisting of repetitive contractions across multiple muscle groups. While causality cannot be established, this case highlights the importance of providing patients with clear exercise instructions and gradual progression strategies when recommending therapeutic exercise programs, particularly because poor conditioning, unfamiliar exercise, and sudden increases in training volume are recognized risk factors for ER [4,5,24,25].

Identification of this patient’s risk factors in the primary care setting was crucial in facilitating expedited workup and treatment. The preserved renal function in this patient upon admission, as supported by his normal creatinine, blood urea nitrogen, and electrolytes, suggests the importance of prompt management in cases of ER. Standard treatment of rhabdomyolysis centers on early aggressive intravenous crystalloid administration. Published reviews recommend prompt volume repletion with normal saline at approximately 200-1000 mL/hour, titrated to urine output, with a target urine output around 200-300 mL/hour [3,21]. In our patient, continuous normal saline at 250 mL/hour with a goal urine output of 200-300 mL/hour for three days fell within this recommended range. This management resulted in progressive improvement in CK and liver enzyme levels over the patient’s four-day hospitalization.

Overall, this case underscores the importance of considering rhabdomyolysis in patients presenting with dark urine or myalgias after initiating a new exercise regimen, even when the activity is perceived as low-intensity. Clinicians should be aware that unsupervised, high-volume exercise regimens targeting the pelvic floor and surrounding musculature may pose a risk of muscle injury when performed without gradual conditioning, particularly in previously inactive individuals. When prescribing pelvic floor exercises for sexual dysfunction or pelvic floor rehabilitation, providers should emphasize gradual progression in frequency and duration, along with adequate warm-up and cool-down periods, particularly for individuals who are sedentary or unfamiliar with targeted muscle training. Additionally, counseling should include the importance of adequate hydration, proper nutrition, and avoiding exercise in excessively hot environments [24,34].

## Conclusions

ER is a rare and potentially life-threatening condition. While more commonly associated with strenuous, high-intensity exercise, ER may also occur in the setting of prolonged unfamiliar exercise involving repetitive contractions of previously unconditioned muscle groups. This case report highlights an unusual presentation of ER following an unsupervised exercise regimen involving pelvic floor, gluteal, and core exercises and expands the spectrum of exercise-related activities that may be associated with this condition. It also demonstrates the importance of identification of risk factors, such as age, sex, race, and lifestyle behaviors that might support early diagnosis and treatment of rhabdomyolysis. This case also demonstrates that ER is a critical diagnosis to include in a differential even in the absence of other risk factors, such as sickle cell, muscle disorders, high-intensity exercise, excessively hot environment, or dehydration. Finally, this case highlights important considerations when recommending exercise to patients. Clinicians should counsel patients to gradually increase activity levels and maintain adequate hydration, nutrition, and environmental safety to help reduce the risk of exercise-related muscle injury. When recommending pelvic floor exercise programs, particularly for previously unconditioned individuals, clinicians should consider providing specific instruction regarding exercise technique, volume, and progression and, when appropriate, referring patients to physical therapy to support safe participation. Greater awareness of this potential association may facilitate earlier recognition and timely management of rhabdomyolysis, ultimately helping prevent serious complications.
